# Yield stability analysis reveals sources of large-scale nitrogen loss from the US Midwest

**DOI:** 10.1038/s41598-019-42271-1

**Published:** 2019-04-08

**Authors:** Bruno Basso, Guanyuan Shuai, Jinshui Zhang, G. Philip Robertson

**Affiliations:** 1 Department Earth and Environmental Sciences, Michigan State University, East Lansing, MI 48824 USA; 2 W.K. Kellogg Biological Station, Michigan State University, Hickory Corners, East Lansing, MI 49060 USA; 3 Great Lakes Bioenergy Research Center, Michigan State University, East Lansing, MI 48824 USA; 4 State Key Laboratory of Earth Surface Processes and Resource Ecology, Beijing Normal University, Beijing, 100875 China; 5 Department Plant, Soil and Microbial Sciences, Michigan State University, East Lansing, MI 48824 USA

## Abstract

Loss of reactive nitrogen (N) from agricultural fields in the U.S. Midwest is a principal cause of the persistent hypoxic zone in the Gulf of Mexico. We used eight years of high resolution satellite imagery, field boundaries, crop data layers, and yield stability classes to estimate the proportion of N fertilizer removed in harvest (NUE) versus left as surplus N in 8 million corn (*Zea mays*) fields at subfield resolutions of 30 × 30 m (0.09 ha) across 30 million ha of 10 Midwest states. On average, 26% of subfields in the region could be classified as stable low yield, 28% as unstable (low yield some years, high others), and 46% as stable high yield. NUE varied from 48% in stable low yield areas to 88% in stable high yield areas. We estimate regional average N losses of 1.12 (0.64–1.67) Tg N y^−1^ from stable and unstable low yield areas, corresponding to USD 485 (267–702) million dollars of fertilizer value, 79 (45–113) TJ of energy, and greenhouse gas emissions of 6.8 (3.4–10.1) MMT CO_2_ equivalents. Matching N fertilizer rates to crop yield stability classes could reduce regional reactive N losses substantially with no impact on crop yields, thereby enhancing the sustainability of corn-based cropping systems.

## Introduction

Reactive nitrogen loss to the environment is one of the most widespread and recalcitrant environmental problems in major crop-producing regions of the world today. It is especially problematic in those areas where N fertilizer is used extensively, such as the United States, China, and Europe^1–4^, and leads to coastal^5^ and surface water^6^ eutrophication, ground water contamination^7^, elevated rates of N deposition from gaseous emissions of NH_3_ and NO_x_^8^, atmospheric greenhouse gas loading^9^, and stratospheric ozone depletion^10^. The ~110 Tg of N fertilizer applied annually to crops^11^, often in excess of plant requirements^3,12,13^, is almost twice that entering the biosphere during pre-industrial times^14^. Future reactive N losses from excessive N fertilizer use will be further exacerbated by rising demands for food and other agricultural products as global population and affluence increase^15^. Solutions to the imbalance between crop N requirements and the regional amount of N fertilizer applied have been elusive, in part because of the difficulty of linking large scale effects to small scale practices^16^.

The pressure on farmers to increase crop yields for greater economic return often leads to excessive N fertilizer application, despite its economic and environmental cost^1,17–19^. In most of the world, fertilizer is applied uniformly at the beginning of a cropping season in anticipation of high yields and efficient use, even though farmers recognize that yields and N use will be neither uniform nor necessarily efficient in any given year, and that fertilizer not taken up by crops will be lost from fields, thereby lowering profits and harming the environment.

Ideally, N application rates should vary across fields to match the well-known variability of crop growth conditions, which are largely a function of soil, weather, and position in the landscape^20,21^. However, applying fertilizer at variable rates across fields (called precision agriculture) is challenging because precision agriculture requires a detailed understanding of subfield variability and the relationship of this variability to weather and crop growth^12^. For more than a decade gains in N fertilizer use efficiency (and associated gains in environmental protection) were hoped to be achieved through precision N management, but that promise has yet to be realized.

In large part this is because current use of variable rate technologies is low even in technologically advanced countries like the U.S. due to the complexity of converting geospatial information on soil and plant status into appropriate crop management information, and as well to low economic returns based on current application practices^22–24^. In 2012 yield mapping was used on about half of U.S. corn and soybean farms but variable rate application technology, whether for seeds, pesticides, lime, or fertilizers, on only 16–26% in aggregate^25^.

Here we provide a novel remote sensing approach to derive fine-scale yield stability classes that respond differentially to N fertilizer based on historical yield performance. Using non-commercial widely available remote sensing imagery, we quantify the spatial and temporal variability of corn and soybean yields based on in-season growth patterns and provide for subfield areas a partial N balance for corn years, calculated as N fertilizer addition less plant harvest N removal. This value, a form of N use efficiency (the fraction of N input harvested as product), sometimes defined as N recovery efficiency or nitrogen partial factor productivity^13,18,19,26–29^, provides a conservative estimate of the amount of N fertilizer not used by the crop and thereby unnecessarily lost from the system, subsequently providing an estimate of the monetary and environmental savings that could be realized by more precise application of N fertilizers using available geospatial technologies at the subfield scale.

## Results

**Figure 1:**
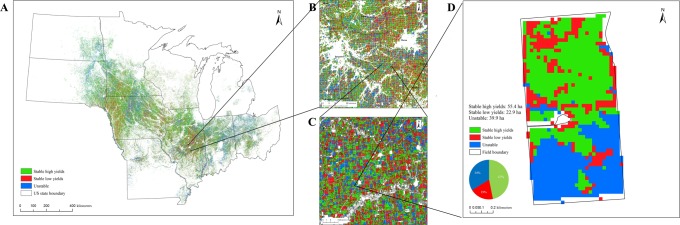
Crop yield stability maps for (**A**) ten U.S. Midwest states and subregions of (**B**) 10,000 km^2^, (**C**) 196 km^2^, and (**D**) 118 ha. Colors represent yield stability areas for 0.09 ha portions of fields planted to corn or corn-soybean for at least three years during 2010-2017 (~30 Mha total).

Figure 1 shows subfield yield stability classes for each field in the region planted with corn or soybean for at least three years of the eight-year study period, including fields planted continuously to corn, to corn-soybean, or (infrequently) more complex rotations. Croplands that did not meet this three-year requirement appear as white pixels in figures. Examination of county and section-level subregions (e.g., Fig. 1B,C, Table 1) shows remarkably high yield variation for a region with yields often considered uniformly stable and high. On average, 46% of the cropland analyzed had stable high yields, 26% stable low yields, and 28% unstable yields (Table 1).

**Table 1:** State-level yield stability trends for 2010–2017.

State	Area (ha)	Percentage of area (%)*			Unstable area yield class (%)	
		Stable high yield	Stable low yield	Unstable yield	High yield	Low yield
Illinois	6,516,484	47 (±7)	30 (±5)	23 (±8)	64	36
Indiana	3,153,424	41 (±7)	25 (±4)	34 (±10)	64	36
Iowa	7,497,549	51 (±8)	31 (±8)	19 (±14)	69	31
Michigan	121,673	38 (±11)	24 (±10)	38 (±17)	64	36
Minnesota	3,894,599	51 (±8)	23 (±6)	26 (±11)	67	33
Missouri	1,414,243	41 (±10)	29 (±8)	30 (±15)	61	39
North Dakota	704,829	50 (±13)	19 (±12)	31 (±14)	67	33
Ohio	1,830,759	42 (±8)	27 (±7)	31 (±13)	62	38
South Dakota	2,064,051	51 (±14)	22 (±11)	28 (±19)	68	32
Wisconsin	867,204	52 (±5)	31 (±3)	16 (±5)	68	32
Average		46	26	28	65	35

*Numbers after ± are the standard deviation values calculated from county-level stability statistics.

There were remarkably few differences among states with respect to the proportion of areas in each class. Stable high yield areas constitute 38–52% of total corn and soybean cropland across these states, with the highest proportions in Wisconsin, Iowa, Minnesota, and the Dakotas (Table 1)—all located in the northwest portion of the region. Likewise, the proportion of land in the low stability class was between 19 and 31% among all states, with unstable areas constituting the remaining 16–38% of total corn and soybean cropland, with the highest proportions in Michigan, Indiana, and Ohio—the easternmost states in the region.

We combined estimates of yield with fertilizer application rates based on the USDA ARMS survey^30^ and university-based recommendations (see Methods). N fertilizer rates from ARMS (Table S1), not including manure inputs, averaged 160 (±24 SD) kg N ha^−1^ yr^−1^ across the 10 states, ranging from 117 to 197 kg N ha^−1^ yr^−1^. These values are self-reported by farmers and lower than university recommendations, and thus provide a conservative, likely minimum fertilizer rate for corn.

University-based N fertilizer recommendations are based on the maximum return to nitrogen (MRTN) database for most states in the region^31^. MRTN rates are ~10% higher than those reported in the ARMS survey and range from 129 to 209 kg N ha^−1^ yr^−1^ (Table 2), for an average rate of 177 (±27 SD) kg N ha^−1^ yr^−1^. Farmers in general^17^ and U.S. farmers specifically^32^ more often use N fertilizer recommendations from fertilizer and seed dealers than from university extension, and when used, MRTN rates are commonly used as starting points for N rate decisions. We thus set our maximum range to 20% greater than local MRTN values to obtain a plausible bracketing.

**Table 2:** N removed by harvest, N fertilizer surplus, and apparent N use efficiency (NUE) within yield stability classes.

State	Fertilizer N Rate	Harvested N			Surplus N			NUE		
		Stable High	Stable Low	Unstable	Stable High	Stable Low	Unstable	Stable High	Stable Low	Unstable
IL	179–229	145	108	131	34–84	71–121	48–98	63–81	47–60	57–73
IN	168–251	135	99	122	33–116	69–152	46–129	54–80	39–59	49–73
IA	152–208	148	115	138	4–60	37–93	14–70	71–97	55–76	66–91
MI	143–198	131	95	118	12–67	48–103	25–80	66–92	48–66	60–83
MN	154–212	145	115	135	9–67	39–97	19–77	68–94	54–75	64–88
MO	189–260	118	85	105	71–142	104–175	84–155	45–62	33–45	40–56
ND	137–190	111	83	102	26–79	54–107	35–88	58–81	44–61	54–74
OH	155–234	136	100	122	19–98	55–134	33–112	58–88	43–65	52–79
SD	134–182	118	89	109	16–64	45–93	25–73	65–88	49–66	60–81
WI	111–155	132	100	122	0–23	11–55	0–33	85–119	65–90	79–110
Total Average	152–212	132	99	120	22–80	53–113	33–92	63–88	48–66	58–81

Values are for corn in stable high yield, stable low yield, and unstable yield areas by US state. All values are kg N ha^−1^ y^−1^ except NUE is kg grain N kg^−1^ N fertilizer.

Using our remotely sensed based crop yields and estimated N fertilizer rates, we calculated average annual N uptake, NUE, and surplus N loss for each stability class (Table 2). Estimated reactive N losses (surplus N) from stable high yield areas ranged from none (indicating the partial use of another source of N such as manure or residual N fixed by soybeans in corn-soybean rotations) to 142 kg N ha^−1^ y^−1^. Annual N losses from stable high yield areas averaged only 51 kg N ha^−1^ (Table 2). In contrast, estimated average N losses from stable low yield areas were 83 kg N ha^-1^ (63% higher), and unstable yield areas had intermediate N losses of 63 kg N ha^−1^. NUE varied correspondingly (Table 2), from an average of 76% for high yield areas to 57% for low yield areas. In unstable subfield areas NUE averaged 70%.

We estimate that over-fertilization of stable low yield areas every year and of unstable areas in low yielding years costs farmers in the region ~USD 485 (267–702) million per year in unused N fertilizer lost to the environment (Table 3). The global warming impact of this N is also significant: Based on the CO_2_ cost of N fertilizer manufacturing plus direct^9^ and indirect^33^ nitrous oxide (N_2_O) emissions from the N fertilizer applied—one of the largest sources of global warming impact in cropping systems^34^—we estimate that 6.8 (3.4–10.1) MMT of CO_2_ equivalents are emitted to the atmosphere from excessive N fertilization (Table 3).

**Table 3:** Surplus fertilizer N loss from stable and unstable low yield areas and its monetary value, embedded energy, and associated CO_2_-equivalent emissions.

State	Surplus N Loss (Gg y^−1^)	Monetary Value (million USD y^−1^)	Embedded Energy (10^6^ GJ y^−1^)	CO_2eq_ Emissions (Mt y^−1^)
IL	192–380	80.6–159.5	13.4–26.6	1.2–2.3
IN	88–241	36.9–101.1	6.1–16.9	0.5–1.5
IA	87–304	36.6–127.6	6.1–21.3	0.5–1.8
MI	6–17	2.3–7.0	0.4–1.2	0.0–0.1
MN	57–204	23.9–85.8	4.0–14.3	0.3–1.2
MO	72–132	30.3–55.6	5.0–9.3	0.4–0.8
ND	35–85	14.7–35.7	2.6–5.9	0.2–0.5
OH	40–133	16.7–55.8	2.8–9.3	0.2–0.8
SD	51–134	21.3–56.1	3.5–9.3	0.3–0.8
WI	8–43	3.6–18.1	0.6–3.0	0.1–0.3
Total Average	1155 (636–1673)	485 (267–702)	78.8 (45–113)	6.8 (3.5–10.1)

Embedded energy refers to the energy cost of producing surplus N. CO_2_-equivalents are greenhouse gas emissions during fertilizer manufacture plus nitrous oxide emissions from applied fertilizer. The N fertilizer price used in this study is the 2017 price of 210 USD/MT.

All told, then, we estimate (Table 3) that ~1155 (636–1673) Gg N is unnecessarily lost from low yielding stable plus low yielding unstable areas in the region annually. Losses would be even higher in years with very unfavorable growing conditions, such as 2012 when most unstable areas had low yields. At a small watershed scale, county-level surface water nitrate concentrations from USGS^6^ appear well correlated with N loss patterns predicted by crop stability classes (Fig. S5).

## Discussion

Stable high yield subfield areas made up 46% of corn-soybean cropland in the 10 Midwest states analyzed here and appear to utilize fertilizer N much more efficiently than low yield areas, which appeared incapable of supporting crop growth at a level sufficient to utilize the majority of the N applied. As a result, stable low yielding areas appeared to contribute most of the reactive N lost to the environment (~44%), with another 31% lost from unstable yield areas in years with low yields (Tables 1, 2, S2). The cost of this lost N is substantial, both to farmers in terms of the monetary value of unused fertilizer N and to society in terms of reactive N that pollutes aquifers, inland and coastal surface waters, and as well adds to the atmosphere’s greenhouse gas burden.

All states had a similar proportion of stable high yield subfield areas (38–52%) and stable low yield areas (19–31%), though there appeared to be a greater percentage of unstable areas in eastern states. This greater percentage may be due to a greater proportion of fields in eastern states with shallower soils and rolling terrains, which create greater dependence on rainfall amounts and distributions compared to prairie states like Iowa and Minnesota, which in general have deeper soils and greater soil water availability. Unstable areas were high yielding 65% of the time, on average (Table 1).

Nitrogen fertilizer recommendations based on yield stability class could thus provide substantial N savings. How practical might such recommendations based on crop yield stability classes be in reality? Non-commercial satellite imagery sufficient to generate NDVI-based yield stability maps is available for most if not all fields in the U.S., and most grain farmers also have access to harvest combine monitors that can produce annual yield maps for individual fields with even greater precision than satellite imagery. Once stable low yield areas are identified, N fertilizer rates could be adjusted downward to match consistently low crop yields in those areas and thereby reduce both economic costs and environmental harm^35,36^. Variable rate technology for N fertilizer application is readily available on commercial market^24^. In unstable yield areas, a modeling-based strategy that, at the time of fertilization, incorporates recent and projected weather could be used to better match N fertilizer rates to that year’s expected crop needs mid-season^36^.

Our estimated cost savings for avoided fertilizer use covers only the monetary value of lost fertilizer, and thus represents only one facet of a full economic analysis that must also include, at the farm scale, the costs of equipment, labor, and information handling, and at the societal scale, the costs of environmental degradation and mitigation. The absolute economic savings, while likely significant, are thus uncertain.

However, our analysis applies only to N fertilization. Performing this analysis for other inputs such as phosphorus, lime, seeds, herbicides, and labor may show that stable low yielding areas are not only environmentally vulnerable but are also economically unprofitable^35,36^. In such cases it may be that these areas are better planted to conservation strips^37^ or, in the future, to perennial biofuel crops^38–40^.

There are several other sources of uncertainty in our analysis. Perhaps the greatest is our estimate of farmer N fertilizer use. Because there are no verifiable sources of reliable N application rates in the U.S., we used a range bracketed by the ARMS survey of self-reported rates for minimum likely values and university recommended rates plus 20% for likely maximum values. We believe this provides a reasonable, conservative estimate of likely minimum and maximum fertilizer rates used by farmers. Estimates of NUE (Table 2), however, suggest that even these fertilizer estimates are too conservative insofar as our NUE estimates are higher than those reported for field studies^26^. If so, then we might expect NUE for low yielding areas to be <50%, with correspondingly higher N losses. The relative importance of different yield stability classes, however, should remain unchanged.

Another source of uncertainty is our assumption that farmers apply N fertilizer at similar rates to all fields cropped similarly. Direct evidence for this is lacking, but we infer that this is mostly the case from very low coefficients of variation for farmer-reported N fertilizer rates in the ARMS survey^30^ (Table S1).

And finally, uncertainties in yield stability validation, while low (Fig. S1), stem from mismatched resolutions between yield monitor data (2 m) and our satellite-based approach (30 m), and by the fact that much more spatial variation is captured using high-resolution yield monitor data from harvesters at their finer resolution. The time interval between the availability of NDVI imagery and yield monitor data can introduce additional error.

Multiple approaches are needed to address the widespread problem of excess agricultural N in the environment^1,3^. Within^41–43^ and edge of field^37,44,45^ remediation measures as well as 4R type approaches^46^ to improving fertilizer use efficiency offer promise for reducing the environmental burden of agricultural N use. Variable rate N fertilizer application based on subfield yield stability as described here provides an additional, nonexclusive means for meeting environmental goals thus far difficult to achieve.

## Methods

### Cropland data layers (CDL)

CDLs are annual raster-format land-use maps created by the USDA National Agricultural Statistics Service (NASS) (www.nass.usda.gov/Research_and_Science/Cropland/Release/index.php). In 2006, CDLs had a spatial resolution of 56 m and land-use categories were based on the Landsat 5 TM, Landsat 7 Enhanced Thematic Mapper (ETM+), the Indian Remote Sensing RESOURCESAT-1 (IRS-P6), and Advanced Wide Field Sensors (AWiFS). Since 2008, the CDLs utilized Landsat TM/ETM + and AWiFS imagery for production of a 30 m product covering the continental US. For our study period CDLs were processed using the Albers Equal-Area Conic Projection with the North American Datum 1983 (NAD83); we re-projected from Albers to the dominant Universal Transverse Mercator (UTM) zone with a spheroid and datum of World Geodetic System 1984 (WGS84). We used CDL data to extract fields in the study area that grew either corn or soybeans.

### Satellite Data

We acquired Google Earth Engine Landsat 5, 7, and 8 images (30 m resolution) between 2010 and 2017 that were consistent with CDL data. The projection of Landsat imagery is dominant UTM with a datum of WGS 84. In 2010 and 2011, Landsat 5 data were preferred due to the SLC failure in the Landsat 7 images. In 2012, only Landsat 7 was used, and the gaps were filled by applying a medium-kernel to the Landsat 7 SLC-off images. For 2013–2017, Landsat 8 data was used as the main source, supplemented by Landsat 7 images. For each growing season, Landsat images during the last two weeks of July were preferred to represent variation in crop growth as there is a high correlation between NDVI and crop yield during this period^47^. While other satellite imagery is also available, NASA Landsat provides the longest continuous quality-assured record at the appropriate scale for subfield analysis.

Areas with cloud cover were replaced with clear pixels from images collected during adjacent periods. Two thresholds were applied to identify cloudy pixels: one threshold of 0.2 was applied to the near infrared (nir) band and another threshold of 1 to the simple cloud-likelihood score image, an internal index in Google Earth Engine derived using a combination of brightness, temperature, and normalized difference snow index (NDSI). After replacing cloudy areas, one composite Landsat image covering ten states was created for each year. The NDVI image was then calculated using Red and Nir bands of this composite image^48^.

### Common Land Unit (CLU)

The CLU data layer is a standardized Geographic Information System (GIS) layer that characterizes the smallest unit of land with a permanent contiguous boundary and common land cover and management. The CLU was established by the USDA Farm Service Agency (FSA) to map the nation’s farm fields, rangeland, and pastureland at a confidence level of 90% with a tolerance of 3 m from ground features visible in the imagery (https://datagateway.nrcs.usda.gov/). This layer is used to implement farm service programs such as crop monitoring, insurance, and disaster assistance. We used the CLU polygons as the spatial boundary for within-field yield variability analysis. The spatial reference of the CLU layer is UTM dominant zone with a datum of NAD83. We converted the original NAD83 datum into WGS84 to keep consistent with other data sources.

### NASS statistics

County-level corn and soybean yields were obtained by USDA NASS^49^, and farmer-reported N application rates to corn were provided by the ERS ARMS^30^.

### N fertilizer rate

Nitrogen fertilizer application rates for individual fields are not directly represented in USDA or other databases. We thus estimated application rates by two independent measures. First are rates reported by farmers in the 2016 USDA Agricultural Resource Management Survey (ARMS) of corn farmers^30^. This survey, conducted on a 5-year cycle for each major commodity, is based on in-person interviews with farmers (1209 farmers in the 10 states covered in our analysis), and we use reported average rates, with variation among rates within states to provide a measure of uncertainty, for all fields within a county.

The second measure was constructed from university-based recommendations. For six states in the region university extension recommendations are based on the Maximum Return to Nitrogen (MRTN) calculator (http://cnrc.agron.iastate.edu/About.aspx)^31^. The MRTN calculator determines an average economically optimal corn N fertilizer rate for fields within IL, IA, MI, MN, OH, and WI based on thousands of research trials conducted for corn following corn and corn following soybean rotations. Because calculator values are generally used to define a baseline value for any given field, with actual fertilizer recommendations rates usually greater than MRTN calculated values, the calculator provides a second conservative estimate of fertilizer use in the region.

We used the MRTN calculator to estimate optimal fertilizer rates using a common 1:10 price ratio of corn and N fertilizer (USD 4/bu and USD 0.42/lb, respectively, equivalent to USD 157/MT and USD 210/MT, respectively)^31^. For states where MRTN data were not available, we used ARMS rates plus 10.3%, the average difference between MRTN and ARMS rates for all states with both data available. We held the amount of applied N constant from 2010 to 2017.

Crop residues are assumed to be recycled internally and to not accumulate or increase SOM, thus they are not considered here.

### NDVI Stability Classes

Crop yield stability classes have long been used to create management zones within the field based on inter-annual yield variation^47,50–53^. Typically, data are collected from georeferenced yield monitor systems mounted on harvesters, with yield stabilities resolved to few m^2^.

We estimated yield stabilities in the absence of high-resolution yield monitor data by analyzing year-to-year variability of satellite-derived NDVI for 0.09 ha subfield areas within individual fields. We used eight years of available imagery and CDL data to determine how a given 30 × 30 m subfield pixel changed from year to year relative to the mean NDVI for the entire field. This analysis was performed to determine areas that had, over the studied years, (1) consistently higher NDVI compared to the mean NDVI of the field (high & stable yields, SH), (2) a consistently lower NDVI (low & stable yields, SL), or (3) an inconsistent (lower some years, higher others) NDVI (unstable yields, U).

For the *i*th year, the average NDVI value of the *j*th CLU polygon was calculated and all pixels within this polygon were classified into two types (higher than average of the field and lower than average of the field for a given year):

1 $${\overline{NDVI}}_{i,j}=\frac{\sum _{1}^{n}NDV{I}_{i,j,k}}{n}$$

2 $$rNDV{I}_{i,j,k}=\frac{NDV{I}_{i,j,k}-{\overline{NDVI}}_{i,j}}{{\overline{NDVI}}_{i,j}}$$

3 $$snNDV{I}_{i,j,k}=\{\begin{array}{c}High\,NDVI\,if\,NDV{I}_{i,j,k}\,\ge {\overline{NDVI}}_{i,j}\\ Low\,NDVI\,if\,NDV{I}_{i,j,k}\, < {\overline{NDVI}}_{i,j}\end{array}$$

where *n* and *k* indicate the total number of pixels and *k*th pixel in the *j*th polygon. *rNDVI* is relative NDVI, and *snNDVI* is the spatially normalized NDVI for a given year.

We then determined the temporal variation of snNDVI expressed as degree of stability, or tnNDVI (temporally normalized NDVI). We calculated year-by-year variation for each corn and soybean pixel and then classified each pixel as stable high, stable low, or unstable NDVI, using the following equations:

4 $${\overline{snNDVI}}_{j,k}=\frac{\sum _{1}^{8}snNDV{I}_{i,j,k}}{8}$$

5 $$tnNDV{I}_{j,k}=\sqrt{\frac{1}{8}\sum _{1}^{8}{(snNDV{I}_{i,j,k}-snNDV{I}_{j,k})}^{2}}$$

6 $$Stabilit{y}_{j,k}=\{\begin{array}{c}SH\,if\,NDV{I}_{i,j,k}\,\ge {\overline{NDVI}}_{i,j}\,and\,tnNDV{I}_{j,k} < 0.15\\ SL\,if\,NDV{I}_{i,j,k}\,\ge {\overline{NDVI}}_{i,j}\,and\,tnNDV{I}_{j,k} < 0.15\\ U\,if\,tnNDV{I}_{j,k}\ge 0.15\end{array}$$

Stable high NDVI pixels were identified where the pixelwise NDVI over the eight years was always greater than the average of the field with a tnNDVI less than 0.15. Stable low NDVI pixels were identified where the mean NDVI for each pixel for the eight years was always lower than the average of the field with a tnNDVI less than 0.15. Unstable areas were identified where the tnNDVI was greater than 0.15.

### Yield and N Uptake Estimates

We estimated subfield yields by deconvoluting observed USDA NASS county-level yields for any given year into high and low yield areas using equations (1–3) based on their respective NDVI values to maintain the proportionality of differences among NDVI using the following equations:

7 $$Yiel{d}_{i,j}=Are{a}_{high,i,j}\times Yiel{d}_{high,i,j}+Are{a}_{low,i,j}\times Yiel{d}_{low,i,j}$$

8 $$\frac{Yiel{d}_{high,i,j}}{Yiel{d}_{low,i,j}}=\frac{{\overline{NDVI}}_{high,i,j}}{{\overline{NDVI}}_{low,i,j}}$$

where $Are{a}_{high,j}$ and $Are{a}_{low,j}$ indicated the acreage of high and low NDVI areas, respectively. ${\overline{NDVI}}_{high,j}$ and ${\overline{NDVI}}_{low,j}$ are mean NDVI values for area. We directly assigned the county-level yield to $Yiel{d}_{i,j}$ based on the assumption that summed crop yields for all fields within a county are equal to the county-level yield.

Grain N uptake (*NUP*) was determined by multiplying yield by grain N percent (1.2%)^54,55^. Residues are assumed to be retained on the soil and decomposed. NUE, defined as kg grain kg^−1^ N applied assuming no change in total soil N^26^, was derived by dividing grain N uptake (*NUP*) by N applied (${N}_{app}$).

9 $$NUE=NUP/{N}_{app}$$

### Nitrogen Balance Calculations

Estimated N loss for a given subfield area was calculated as the difference between N fertilizer applied and grain N output for a given year^3,13,19^. Grain N output was calculated as yield × N content; modern grain varieties have a remarkably consistent grain N content of 1.2%^54,55^.

Our partial N calculation assumes stable internal stores of N (as soil organic matter including crop residue), since any increase in internal storage might otherwise be mis-attributed to N loss. Soil organic matter is known to be stable or declining across the US Midwest except where permanent no-till or cover crops are present (low single digit percentages of cropland^56–58^), so we expect no regional changes in soil organic matter that would significantly reduce our loss estimates. Our calculation also assumes there are no significant changes in other N inputs across the region. Nitrogen inputs additional to fertilizer in these systems include atmospheric N deposition and N fixed by a prior soybean crop. We do not include either in our analysis because both are negligible (<10 kg N ha^−1^ yr^−1^) in comparison to the amount of N fertilizer used, and their inclusion would in any case increase rather than decrease our estimates of N loss. Thus their exclusion makes our environmental loss term even more conservative.

We do not differentiate between hydrologic and gaseous losses of N except to estimate the amount of nitrous oxide (N_2_O) emissions (both direct and indirect) from added fertilizer, calculated using IPCC emission factors^59^.

### Monetary value and Environmental losses

Direct monetary value and environmental losses were calculated as:

10 $$USD\,Loss\,(USD/ha)=0.42\,USD/kg\times NLoss$$

11 $$Energy\,Loss\,(MJ/ha)=70MJ/kg\times NLoss$$

12 $$C{O}_{2}\,equivalent\,(kg/ha)=NLoss\times 6.04$$

The monetary value of lost fertilizer N is based on the 2017 fertilizer value of USD 210 USD/MT (0.42 USD per kg N). The coefficients used in equation 11 and 12 were obtained from^59,60^.

### Yield Stability Validation

We verified yield estimates (at 30 m resolution) for corn and soybean against data from combine harvester monitors (at 2 m resolution) for 508 corn fields across the region (Fig. S1–4). Most fields were in a corn and soybean rotation for at least 3 years. Yield data were collected from combine harvesters equipped with yield monitors and recorded as point data at a 2 m interval for each row. Stability maps were generated from yield monitor data using the same procedure as for NDVI images. Comparisons of our calculated yield stability classes vs. high resolution yield monitor data for the 508 fields are given in Figs S1–S3.

## Supplementary information

Supplementary Information
