# Supplementary material for: Yield stability analysis reveals sources of large-scale nitrogen loss from the US Midwest
Source: Sci Rep. 2019 Apr 8;9:5774. doi: 10.1038/s41598-019-42271-1 (PMC6453884; doi:10.1038/s41598-019-42271-1)

Yield stability analysis reveals sources of large-scale nitrogen loss from the US Midwest

Bruno Basso[1,2,3*], Guanyuan Shuai[1], Jinshui Zhang[4,1] and G. Philip Robertson[2,3,5]

[1] Dept. Earth and Environmental Sciences, Michigan State University, East Lansing, MI 48824, USA.

[2] W.K. Kellogg Biological Station, Michigan State University, Hickory Corners, MI 49060, USA.

[3]Great Lakes Bioenergy Research Center, Michigan State University, East Lansing MI 48824 USA.

[4]State Key Laboratory of Earth Surface Processes and Resource Ecology, Beijing Normal University, Beijing 100875, China.

[5]Dept. Plant, Soil and Microbial Sciences, Michigan State University, East Lansing, MI 48824, USA.

*E-mail: basso@msu.edu

Supplementary Information

Table S1. Average state-level N fertilizer rates and Coefficient of Variations (CV, %) as reported by ARMS (NASS 2016) and University-recommended rates based on statewide Maximum Return to Nitrogen (MRTN) databases (Sawyer et al. 2006). All values are kg N ha^{-1} y^{-1}.

State	ARMS ± CV (%)	MRTN
IL	183 ± 2%	191
IN	175 ± 4%	209
IA	158 ± 4%	173
MI	151 ± 5%	165
MN	160 ± 4%	177
MO	197 ± 4%	217
ND	143 ± 4%	158
OH	174 ± 11%	195
SD	138 ± 3%	152
WI	117 ± 5%	129

*https://www.nass.usda.gov/Quick_Stats. (2016)

Table S2. Average N fertilizer surplus and percent contribution from each yield stability classes.

State	Average Fertilizer N Rate	Average Surplus N			Average Contribution to Surplus N		
		Stable High	Stable Low	Unstable	Stable High	Stable Low	Unstable
IL	204	59	96	73	26	42	32
IN	210	75	111	88	27	41	32
IA	180	32	65	42	23	47	30
MI	171	40	76	53	24	45	31
MN	183	38	68	48	25	44	31
MO	225	107	140	120	29	38	33
ND	164	53	81	62	27	41	32
OH	195	59	95	73	26	42	32
SD	158	40	69	49	25	44	31
WI	133	12	33	17	19	53	27
Total Average	182	52	83	63	25	44	31

Values are for corn in stable high yield, stable low yield, and unstable yield areas by US state. All values are kg N ha^{-1} y^{-1} except percentage (%) of average surplus N contributed from each area.

Figure S1. Estimated yield using NDVI-stability class approach vs yield monitor data (right-hand bars).

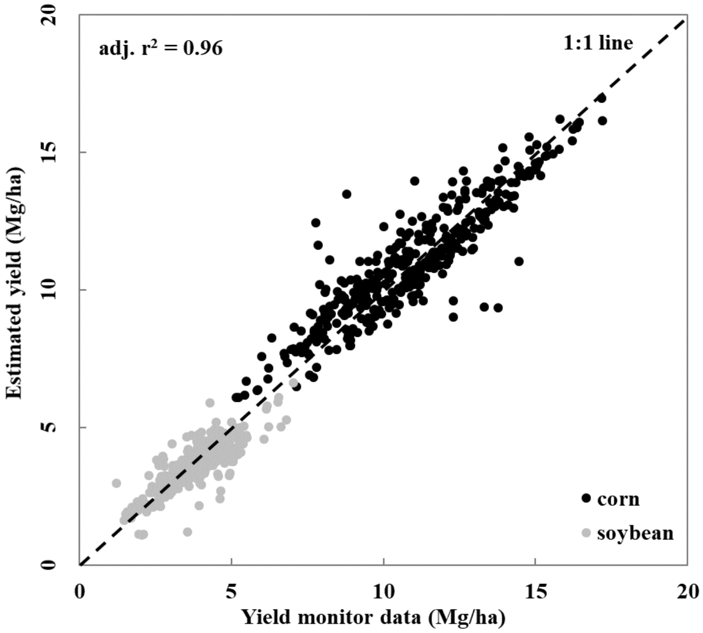

Figure S2. The acreage percentages of stable high yield, stable low yield, and unstable yield areas of Iowa calculated from yield monitor combine harvesters (left-hand bars) or NDVI data (right-hand bars).

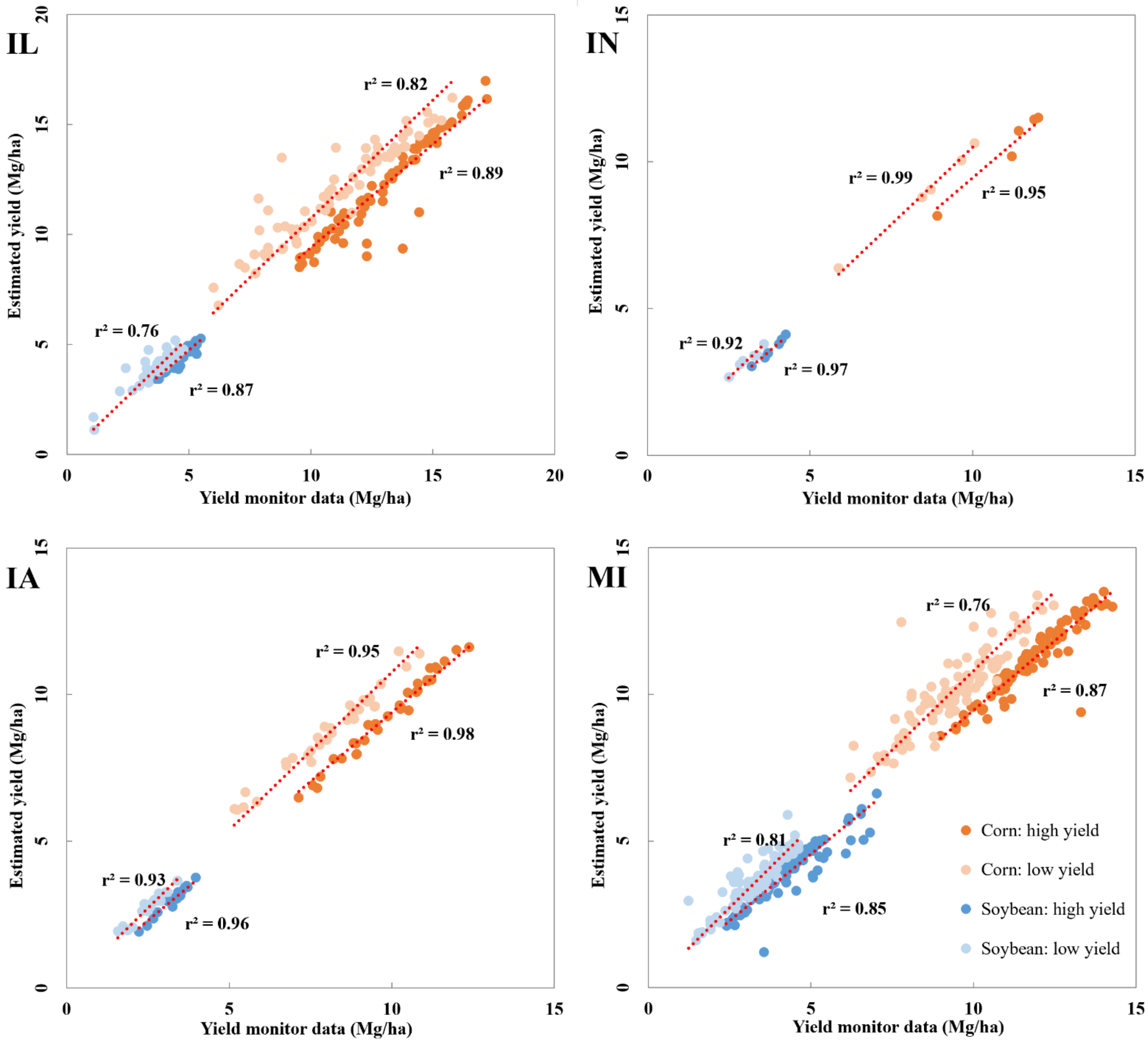

Figure S3. The acreage percentages of stable high yield, stable low yield, and unstable yield areas of Iowa calculated from yield monitor combine harvesters (left-hand bars) or NDVI data (right-hand bars).

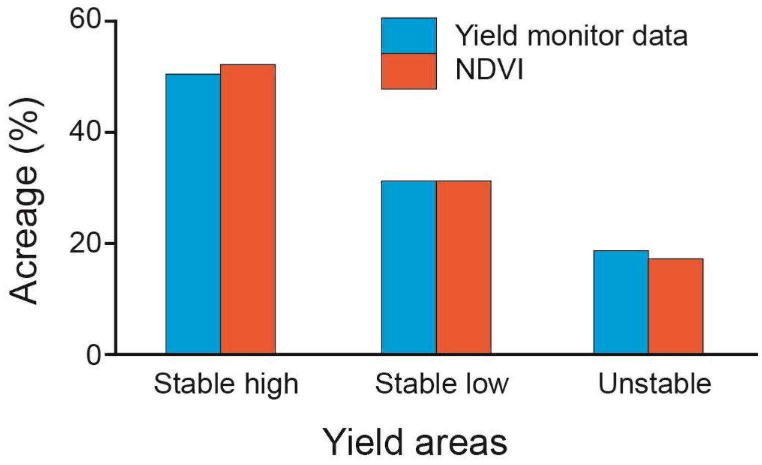

Figure S4. Yield stability class areas derived from combine-based yield monitor data (left-hand panels) and LANDSAT NDVI data (right-hand panels) for two representative Iowa fields (top and bottom rows). The LANDSAT NDVI spatial resolution is 30 m; yield monitor resolution is 2 m.

Yield monitor -based

Landsat -based

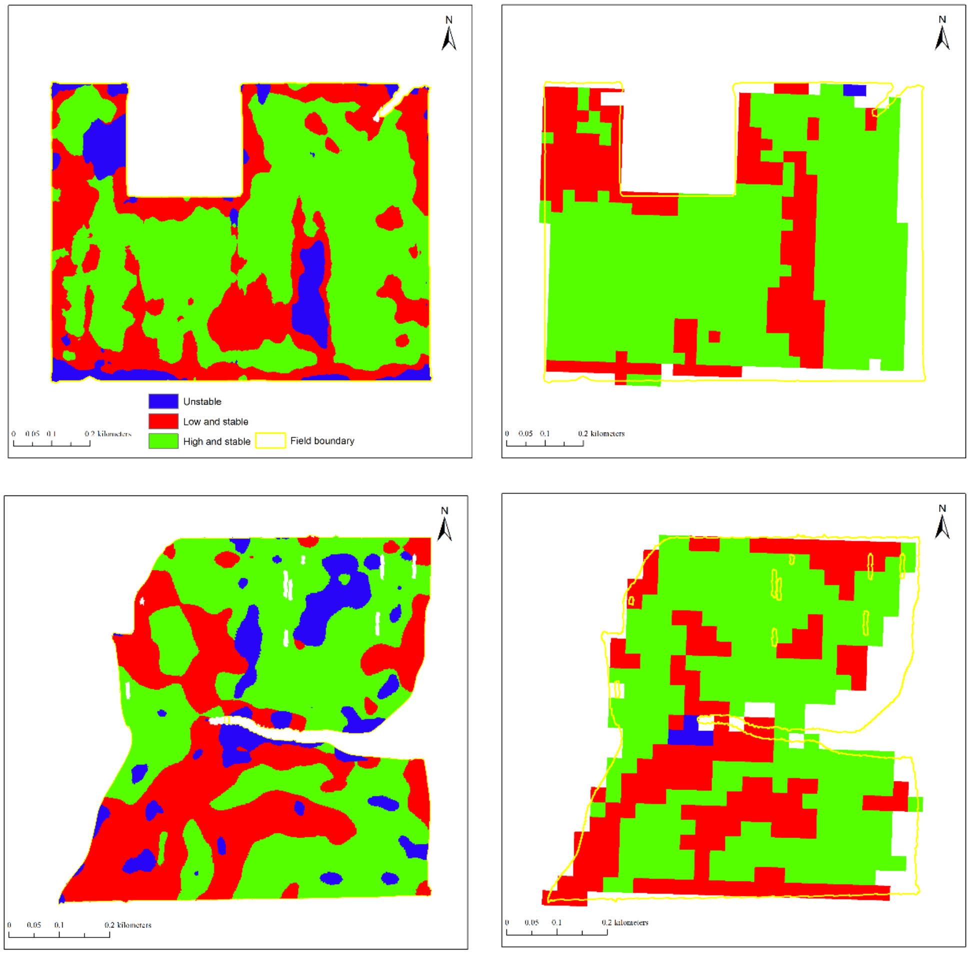

Figure S5. (A) County-level N loss in 2012-13 growing season and (B) mean 2013 nitrate concentration at the 100 Midwest Stream Quality Assessment sites from [6]. The red circles denote areas with concurrent higher N loss and nitrate concentrations. The blue rectangles are areas with lower N loss and nitrate concentration. Reproduced by permission from Peter C. Van Metre et al., 2016.

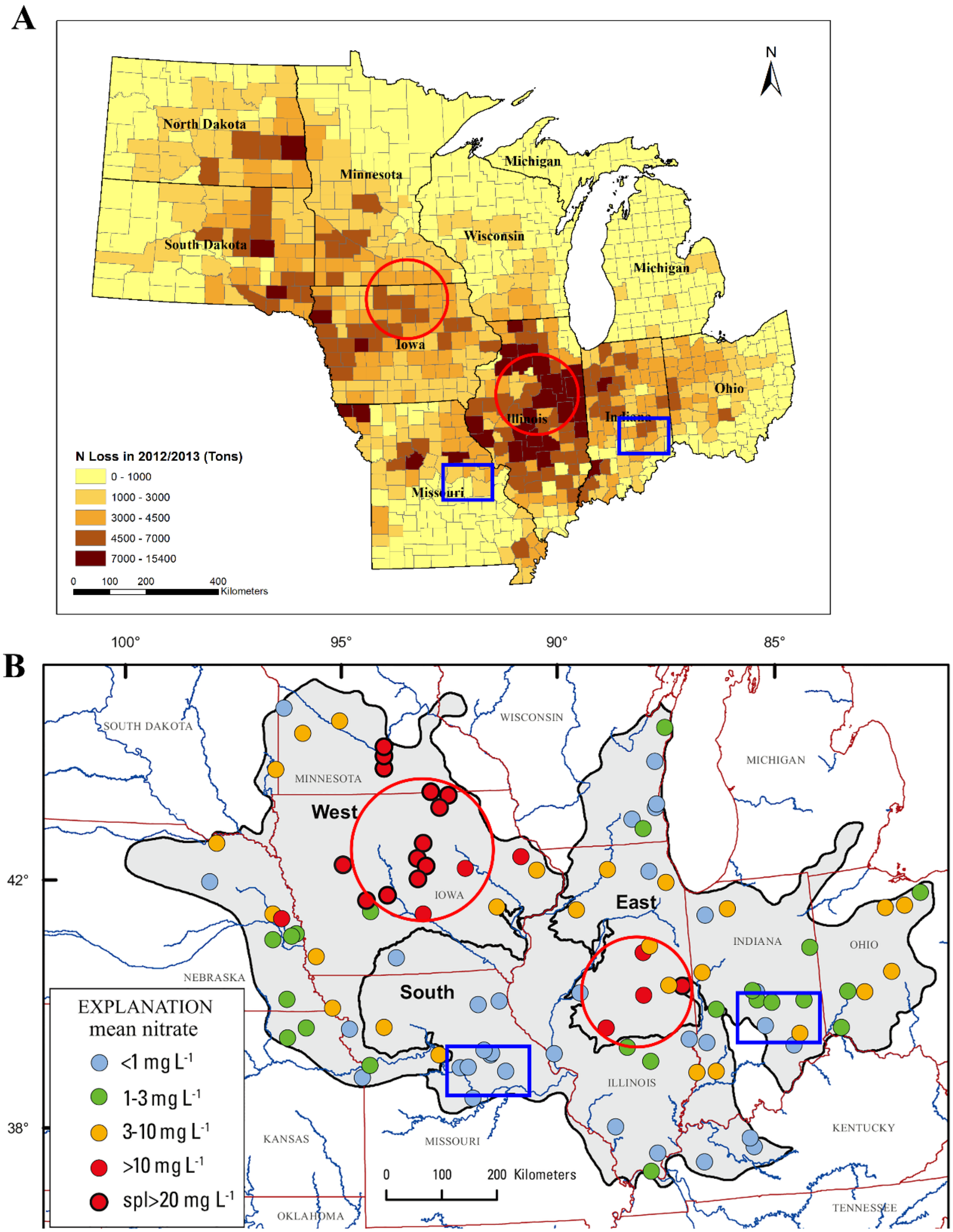

Supplement: Supplementary file 1 — Supplementary Information [file 41598_2019_42271_MOESM1_ESM.pdf]
